# Identification of metabolic dysregulation and biomarkers for clear cell renal cell carcinoma

**DOI:** 10.1002/ctm2.70142

**Published:** 2024-12-26

**Authors:** Bin Zheng, Kan Liu, Qing Ouyang, Ji Feng, Shouqing Cao, Li Wang, Tongyu Jia, ShengPan Wu, Xin Ma, Xu Zhang, Xiubin Li

**Affiliations:** ^1^ Medical School of Chinese PLA Beijing China; ^2^ Department of Urology The Third Medical Centre, Chinese PLA General Hospital Beijing China; ^3^ College of graduate, Hebei North University Zhangjiakou China

1

Dear Editor,

Clear cell renal cell carcinoma (ccRCC) is the most common RCC histology and a metabolic disease characterized by reprogramming of energetic metabolism, enabling cancer cells to proliferate rapidly and survive.^1^ Understanding these metabolic changes may provide opportunities for discovering biomarkers, improving tumour detection and developing new therapeutic strategies.

This study aimed to identify and validate metabolites as biomarkers to improve clinical diagnosis, facilitate risk stratification and predict prognosis in ccRCC. We used targeted metabolomics to screen for metabolic biomarkers and compared the differential metabolites between our data and other six published studies^2^, ^3^, ^4^, ^5^, ^6^ (Table S1). Detailed information on metabolite measurement and methods is provided in the Supporting Information.

In our cohort, 90 tissues (30 from early patients, 30 from advanced patients and 30 from normal tissues) were collected from 60 ccRCC patients (Table S2). Principal component analysis (PCA) and partial least squares discriminant analysis (PLS‐DA) analyses revealed a clear separation between the two groups (Figure S1A,B). The trend of quality control samples and permutated R2, and Q2 values indicated good analytical reproducibility and low risk of overfitting (Figure S1D,E). The differential analysis demonstrated that 163 metabolites (67 metabolites up‐regulated and 96 metabolites down‐regulated) from 23 distinct classes were significantly altered in tumour tissues (Figure 1A–F and Table S3). Then, we divided patients into early and advanced cohorts to perform differential analyses. The PCA and PLS‐DA analyses showed distinct differences (Figure S1B,C). 79 metabolites were significantly different between early tumour and paired normal tissues (39 metabolites up‐regulated and 40 metabolites down‐regulated) (Figure 1G and Table S3), and 64 metabolites significantly distinguished advanced tumour from paired normal tissues (25 metabolites up‐regulated and 39 metabolites down‐regulated) (Figure 1H and Table S3). The pathway enrichment analysis of differential metabolites revealed eight intersected KEGG pathways. Two metabolic processes were substantially enriched in the early‐stage cohort, while three processes were enriched in the advanced‐stage cohort (Figure 1I,J and Table S4).

**FIGURE 1:**
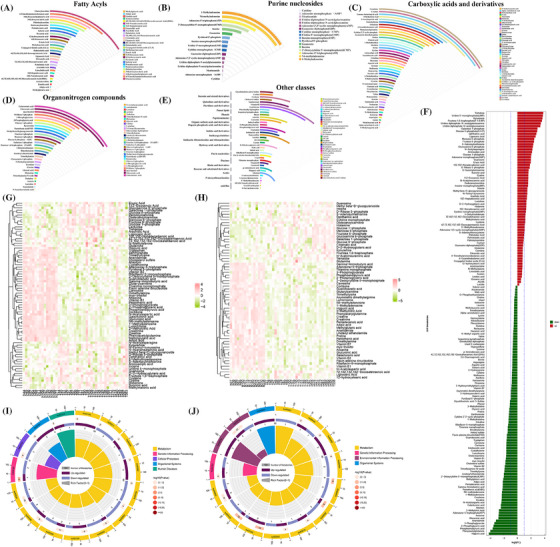
The expression characteristics of metabolites in clear cell renal cell carcinoma (ccRCC). (A–E) Rainbow plot of differential metabolites. (F) Bar chart for the differential metabolites. (G, H) Heatmap overview of the differential metabolites in early tumour stage (G) and advanced tumour stage (H). (I, J) The pathway enrichment analysis of differential metabolites in early stage (I) and advanced stage (J) patients.

To understand the differences in metabolites between early and advanced stages, we used the unsupervised clustering analysis. Among early patients, two early clusters (A and B) were identified, and patients in early cluster A had the worst prognosis. Similarly, in the advanced cohort, we identified three advanced clusters (A, B and C) and found that patients in the advanced cluster B had the worst prognosis and patients in the advanced cluster C had a better prognosis (Figure 2A–D and Figure S1F–L). Then, we found elevated tryptophan and creatine in early‐cluster B. Similarly, in advanced‐cluster C, high creatine was found (Figure 2E,F). Considering valuable biomarkers are rarely utilized in ccRCC patients, we screened metabolites using random forest analysis. Based on mean decrease accuracy, we selected the top 10 metabolites in the total, early, and advanced cohorts (Figure 2G–I). A receiver operating characteristic (ROC) showed the top 10 metabolites had high predictive accuracy (area under the curve [AUC] > 0.9) in distinguishing patients (Figure 2J–L and Table S5). Then, we combined all 10 metabolites and found the combined model also had high predictive accuracy (AUC > 0.9) (Figure 2M–O). These results showed that these 10 metabolites could be valuable parameters in the prediction of patients.

**FIGURE 2:**
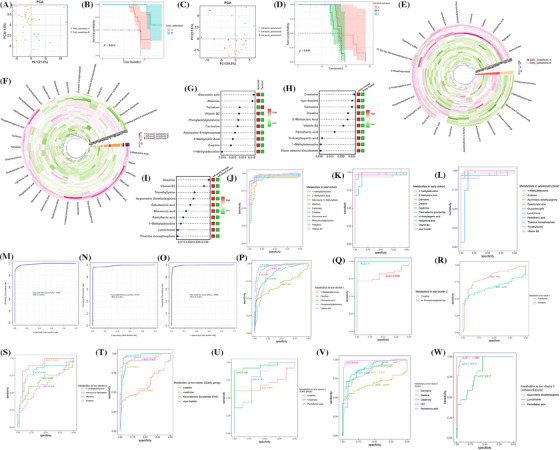
Distinct metabolic clusters and diagnostic potential of metabolites. (A, C) Principal component analysis (PCA) of patients in metabolic clusters based on early (A) and advanced (C) stage renal cell carcinoma (RCC) cohort. (B, D) K–M curve showing the OS for two (B) and three (D) metabolic clusters. (E, F) Differential metabolites between two (E) and three (F) clusters. (G–I) Random forest analysis revealed the 10 most important metabolites in all (G), early (H) and advanced (I) RCC samples. (J–L) Receiver operating characteristic (ROC) curves of the selected metabolites in all (J), early stage (K) and advanced stage RCC cohort (L). (M–O) Combined ROC curve analysis of top 10 metabolites in all (M), early stage (N) and advanced stage RCC cohort (O). (P–S) ROC curves of intersected metabolites between this study and test cluster 1 (Hakimi et al.) (P), test cluster 2 (Li et al.) (Q), test cluster 3 (Hu et al.) (R), test cluster 6 (Reigle et al.) (S) in all RCC cohort. (T–V) ROC curves of intersected metabolites between our study and test cluster 1 (T), test cluster 2 (U) and test cluster 3 (V) in early stage RCC cohort. (W) ROC curves of intersected metabolites between this study and test cluster 3 in the advanced stage RCC cohort.

To further validate the predictive capability, we compared external validation datasets from six independent studies with our data and took the intersection. After excluding two studies without stage information, four validation studies were utilized.^2^, ^3^, ^4^, ^5^ All intersected metabolites had better predictive performance in validation datasets (Figure 2P–W). Although only N‐Phenylacetylglutamine was found in an advanced cohort from test cluster 2, it exhibited good predictive ability (Figure S1J).

Next, we evaluated the role of metabolites in predicting prognosis using external data. We set our data as the training cohort to construct the risk model, and other studies with full clinical information were used as validation cohorts. We first divided our data into total RCC cohort (*n* = 60), early (*n* = 30) and advanced (*n* = 30) RCC cohort. Using lasso analysis, we constructed risk models and found the high‐risk group presented worse outcomes in the total and early RCC cohort (Figure 3A–D). However, there was no significant difference between the two groups in the advanced RCC cohort (Figure 3E). Additionally, the ROC curve demonstrated a strong capacity of the risk model in predicting OS (AUC = 1) in total and early RCC cohorts (Figure 3F, G). These results suggest that the risk model may effectively evaluate the survival of RCC patients. Then, we validated the risk model using the Hakimi et al. dataset. The K‐M curve indicated that the high‐risk groups had worse prognosis in our total and early RCC cohort (Figure 3H,J). However, due to the low sample number, we found although there is a trend between high and low‐risk groups, the difference did not reach statistical significance (Figure 3I,K). Therefore, further validation is still necessary.

**FIGURE 3:**
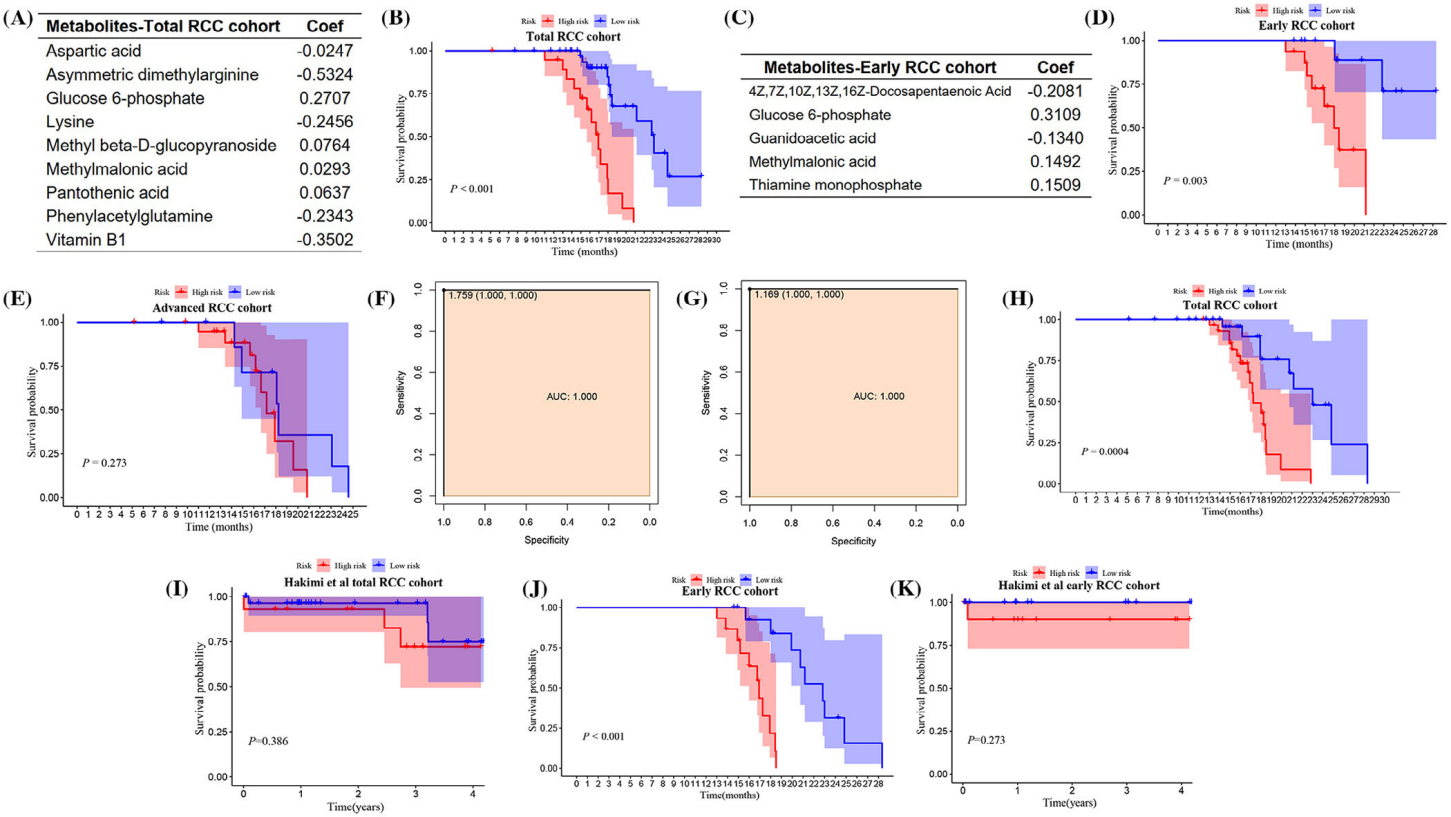
Construction and evaluate the metabolites‐based risk model. (A, C) Metabolites were included in the risk model based on the total (A) group and early (C) stage cohort. (B, D, E) The K‐M analysis for the OS of patients in the high and low‐risk groups among total (B) early (D) and advanced (E) groups. (F, G) Receiver operating characteristic (ROC) curves of training cohort based on total (F) and early (G) patients. (H, I) The K‐M analysis for the OS of total RCC patients among training (H) and test (I) groups. (J, K) The K‐M analysis for the OS of early‐stage RCC patients among training (J) and test (K) groups.

Finally, we compared all differential metabolites between our data and others, to determine whether metabolite alterations were consistent across populations. After comparing metabolomics data in Hakimi et al.,^2^ Hu et al.,^4^ Popławski et al.^7^ and Li et al.^3^ studies, we found the content of most metabolites was consistent with our results. However, creatine, citrulline and inosine content were apparently different (Figure 4A–C and Figure S1M–Y). We further mined their effect on ccRCC. We found creatine was abnormally low in tumour samples (Figure 4D). Then, we explored IC_50_ of creatine and used CCK8 and plate cloning experiments to determine its effect on proliferation, which indicated that creatine (5.28 mM) markedly inhibited RCC cell proliferation (Figure 4E–H). Wound healing assay, Transwell migration and invasion assay demonstrated that creatine significantly inhibited ccRCC cell migration and invasion (Figure 4I–K). Referring to previous research, citrulline and inosine were supplemented at 16.67 mM^8^ and 0.19 mg/ml,^9^ separately. Citrulline suppressed ccRCC cell proliferation, migration and invasion, while inosine promoted RCC cell progression (Figure 4L‐Q). We speculated variations in metabolite concentration may be partially attributed to differences in study samples and contexts.

**FIGURE 4:**
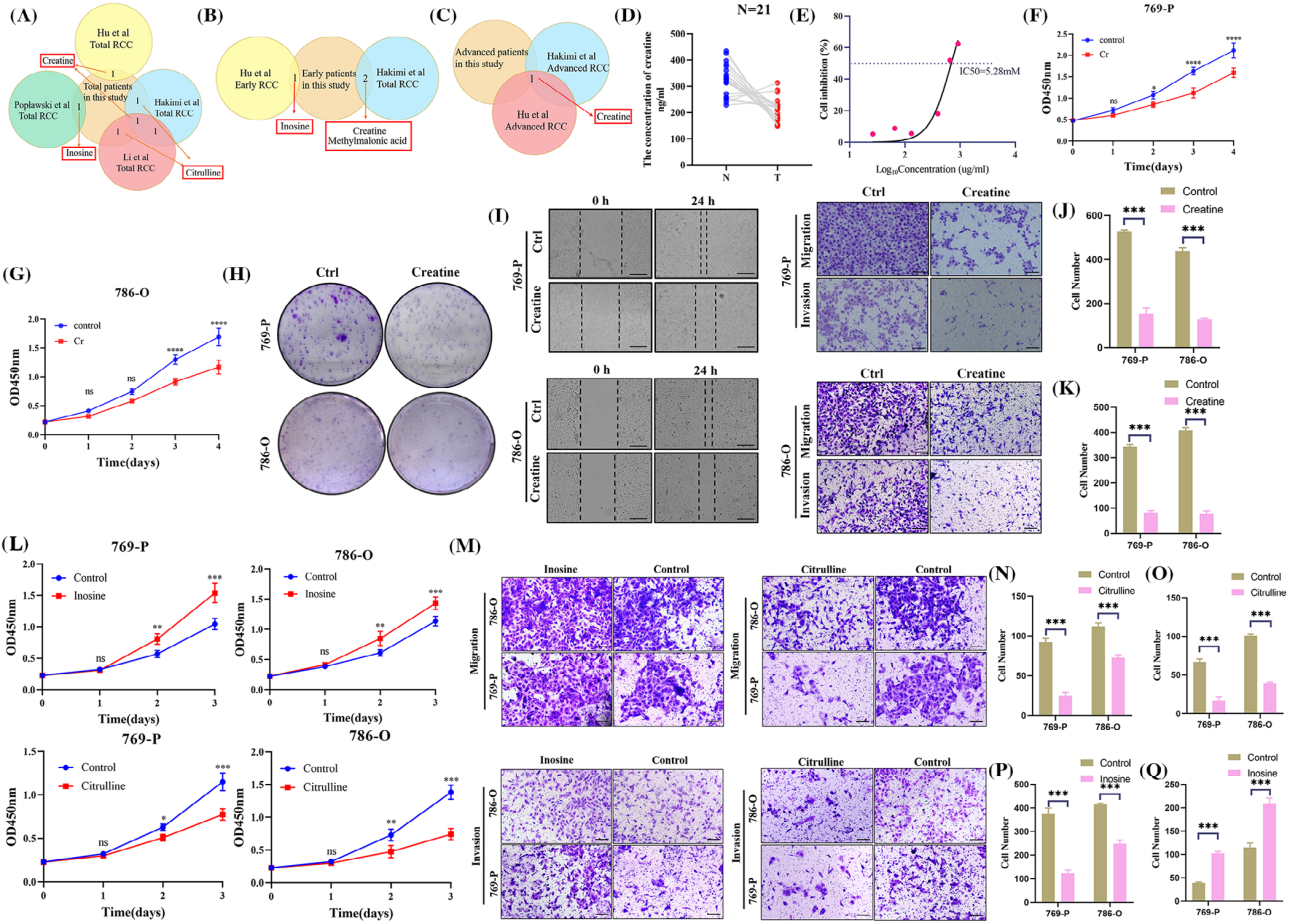
Identification of differential metabolites between diverse studies and their effect on clear cell renal cell carcinoma (ccRCC) cells. (A–C) The intersected differential metabolites in total (A), early (B) and advanced (C) RCC samples. (D) The concentration of creatine in 21 normal samples and 21 tumour samples from ccRCC patients. (E) Identification of IC_50_ of creatine. (F–H) The effect of creatine on ccRCC cell proliferation was assessed by the CCK8 assay (F, G) and plate colony formation assay (H). (I) The effect of creatine on ccRCC cell migration and invasion was measured by wound healing assay (left, scale bar: 400 µm) and Transwell assay (right, scale bar: 100 µm). (J, K) The histogram shows the results of the transwell migration (J) and invasion (K) assay. (L) The effect of inosine and citrulline on ccRCC cell proliferation was assessed by the CCK8 assay. (M) The effect of inosine and citrulline on ccRCC cell migration and invasion was assessed by the Transwell assay. (right, scale bar: 100 µm). (N, O) The histogram shows the effect of citrulline on migration (N) and invasion (O). (P, Q) The histogram shows the effect of inosine on migration (P) and invasion (Q).

Accurate prognostication of oncological outcomes is crucial for ccRCC. Although many gene‐based models exist, metabolite‐based signatures are rare.^10^ We identified key metabolites and constructed a metabolites‐based risk model, which provides predictive and prognostic information independent of clinicopathologic factors. We also evaluated three metabolites, offering insights into the molecular mechanisms and novel therapeutic targets. In short, our study discovered novel biomarkers for diagnosing ccRCC and predicting prognosis at different stages.

## AUTHOR CONTRIBUTIONS

Bin Zheng., Kan Liu., Qing Ouyang. and Xiubin Li. designed research. Bin Zheng., Kan Liu. and, Qing Ouyang. performed research. ShengPan Wu., Li Wang., Tongyu Jia., Shouqing Cao. and Ji Feng contributed reagents and resources. Bin Zheng. and Xiubin Li. wrote the paper in discussion with other co‐authors. Xin Ma. and Xu Zhang. conceived and directed the study.

## CONFLICT OF INTEREST STATEMENT

The authors declare no conflict of interest.

## FUNDING INFORMATION

This work was supported by the National Natural Science Foundation of China (81802804, 82403839), Sino‐German Mobility program (M0735), PLA General Hospital Youth Independent Innovation Science Fund Growth Project (22QNCZ029), PLA General Hospital‐Third Medical Center Discipline Innovation and Development Special Fund Project (2024BJ‐04) and Fostering Fund of Chinese PLA General Hospital for National Excellent Young Scholar Science Fund (2020‐YQPY‐006).

## ETHICS STATEMENT

All studies were approved by the Ethics Committee of the third medical centre of PLA General Hospital.

## Supporting information



Supporting Information

**FIGURE S1** (A) Score plot resulting from principal component analysis (PCA) (left) and PLS‐DA (right) analysis of metabolites in total patients. (B, C) Score plot resulting from PCA (left) and PLS‐DA (right) analysis of metabolites in patients with early‐stage (B) and advanced‐stage (C). (D) The PCA analysis of QC. (E) PLS‐DA cross‐validation plot based on all samples. (F–I) Unsupervised clustering analysis of differential metabolites identified in early‐stage samples (F, G) and advanced samples (H, I). (J) The ROC curve of N‐phenylacetylglutamine. (K, L) Unsupervised clustering of metabolites in early (K) and advanced (L) stage RCC cohort. The metabolic cluster, tumour T stage, survival status, gender, and Fuhrman nuclear grade were used as annotations. (M–R) The intersected differential metabolites in total RCC samples between this study and other independent studies. (S, U, W and X) The intersected differential metabolites in early‐stage RCC samples between this study and other independent studies. (T, V and Y) The intersected differential metabolites in advanced samples between this study and other independent studies.

**TABLE S1** Characteristics of studies included in this study.

**TABLE S2** Descriptive baseline characteristics of 60 patients diagnosed with clear cell carcinoma between 2020 and 2021.

**TABLE S3** Differential metabolites in all, early and advanced stage samples.

**TABLE S4** Intersected KEGG pathways selected by comparing results from each tumour stage.

**TABLE S5** The AUC value of the top 10 metabolites in each tumour stage.

## Data Availability

All the materials generated in this study are available upon reasonable request to the corresponding authors.
