# Supplementary material for: Identification of metabolic dysregulation and biomarkers for clear cell renal cell carcinoma
Source: Clin Transl Med. 2024 Dec 26;14(12):e70142. doi: 10.1002/ctm2.70142 (PMC11670740; doi:10.1002/ctm2.70142)
Supplement: Supplementary file 1 — Supporting Information [file CTM2-14-e70142-s004.docx]

**Methods and Materials**

**Study participants**

A total of 60 participants were recruited at the Chinese PLA general hospital (Beijing, China) between January, 2020 and May, 2021. Study protocols were approved by the Ethics Committee of the Chinese PLA general hospital and written informed consent was obtained from all participants involved in this study. Histopathological diagnosis was confirmed by at least two different pathologists. All clinicopathologic information was collected from clinical records and pathology reports.

**Data collection**

The baseline data were retrieved from medical records. The following baseline clinical and pathologic data were recorded: gender, age at the time of diagnosis, BMI, presence of bloody urine, metastases at diagnosis, tumor location, histological subtype, clinical stage. Patients were also asked whether they had cardiac disease, high pressure, diabetes or smoking. The TNM stage was evaluated by the EAU Guidelines (2024 edition). Patients or families were contacted by phone for survival status until May 1, 2023. To gain a more comprehensive understanding of the correlation between RCC and metabolic reprogramming, we conducted a comprehensive search of PubMed to identify relevant literature between January 1, 2014 and May 15, 2024. All eligible researches were independently determined by two reviewers.

**Sample preparation and LC-MS/MS Analysis**

Normal and tumor tissues were obtained from patients undergoing radical nephrectomy. All tissues were immediately frozen in liquid nitrogen for subsequent experiments. To extract metabolites from tissue samples, cold Methanol, acetonitrile and water were mixed as described^1^. All samples were homogenized and sonicated at 4°C. Then samples were centrifuged at 14,000 g for 20 minutes at 4°C and the supernatant was dried in a vacuum centrifuge at 4°C. All samples were re-dissolved and centrifuged^2^, supernatant was taken during LC-MS analysis. The LC-MS/MS Analyses were performed as described^3,4^. The multiple reaction mode (MRM) method was used for mass spectrometry quantitative data acquisition^5^.

**RCC cell lines and cell culture**

The 786-O and 769-P cell were purchased from ATCC, which were cultured in RPMI-1640 with 10% FBS (Gibco). All cell lines were maintained at 37℃ with 5% CO_2_. Inosine (#HY-N0092, MCE, USA), citrulline (#HY-N0391, MCE, USA) and creatine (#HY-W010388, MCE, USA) were dissolved in PBS. The IC_50_ value was measured as described^6^. Creatine were measured using ELISA kit (#YX-031154H, Shanghai Biotec company).

**In vitro experiments**

Cell proliferation was evaluated by CCK-8 assay and plate clone formation assay. Cells were plated at a density of 1,000 cells in 96-well plates, and CCK-8 was added to each well for 4 days. Absorbance at 450 nm was then assessed. For plate clone formation assay, RCC cells were seeded in 6-well plates at a density of 800 cells per well, and cultured for 7 days. Cell migration and invasion was assayed by Transwell migration assay. 1×10^4^ RCC cells, cultured in serum-free medium and creatine, were added into the upper chamber in a final volume of 100μl. In the lower chamber, 700 μl complete medium was added. After 24h, cells were fixed with 4% PFA for 10 min, followed by 0.1% crystal violet staining solution for 10 min. To prepare wound healing assay, 3 × 10^5^ RCC cells treated with creatine were seeded into 6-well plates and cultured in complete medium. After reaching 90% confluence, a line wound was scratched. Subsequently, RCC cells were cultured in serum-free medium. The wounds were observed at 0 h and 24 h.

**Statistical analysis**

The statistical and pathway analyses were performed by Metaboanalyst 6.0 software^7^. Variability was estimated by calculating the coefficient of variation for quality control (QC). Data were mean filtered to remove variables with very small values, normalized by pareto scaling. ROC curves were performed to characterize the estimates of sensitivity and specificity. Prognostic model and KEGG analyses were constructed using R (version 4.2.3). *P* < 0.05 was considered as statistically significant.

**Reference**

1 D'Amico, D. *et al.* The RNA-Binding Protein PUM2 Impairs Mitochondrial Dynamics and Mitophagy During Aging. *Molecular cell* **73**, 775-787.e710, doi:10.1016/j.molcel.2018.11.034 (2019).

2 Lee, P. *et al.* Targeting glutamine metabolism slows soft tissue sarcoma growth. *Nature communications* **11**, 498, doi:10.1038/s41467-020-14374-1 (2020).

3 Corrado, M. *et al.* Dynamic Cardiolipin Synthesis Is Required for CD8(+) T Cell Immunity. *Cell metabolism* **32**, 981-995.e987, doi:10.1016/j.cmet.2020.11.003 (2020).

4 Lee, C. Y. *et al.* Promotion of homology-directed DNA repair by polyamines. *Nature communications* **10**, 65, doi:10.1038/s41467-018-08011-1 (2019).

5 Lösslein, A. K. *et al.* Monocyte progenitors give rise to multinucleated giant cells. *Nature communications* **12**, 2027, doi:10.1038/s41467-021-22103-5 (2021).

6 Hrustanovic, G. *et al.* RAS-MAPK dependence underlies a rational polytherapy strategy in EML4-ALK-positive lung cancer. *Nature medicine* **21**, 1038-1047, doi:10.1038/nm.3930 (2015).

7 Pang, Z. *et al.* MetaboAnalyst 6.0: towards a unified platform for metabolomics data processing, analysis and interpretation. *Nucleic acids research*, doi:10.1093/nar/gkae253 (2024).
