# Supplementary figures and images for: Identification of metabolic dysregulation and biomarkers for clear cell renal cell carcinoma
Source: Clin Transl Med. 2024 Dec 26;14(12):e70142. doi: 10.1002/ctm2.70142 (PMC11670740; doi:10.1002/ctm2.70142)

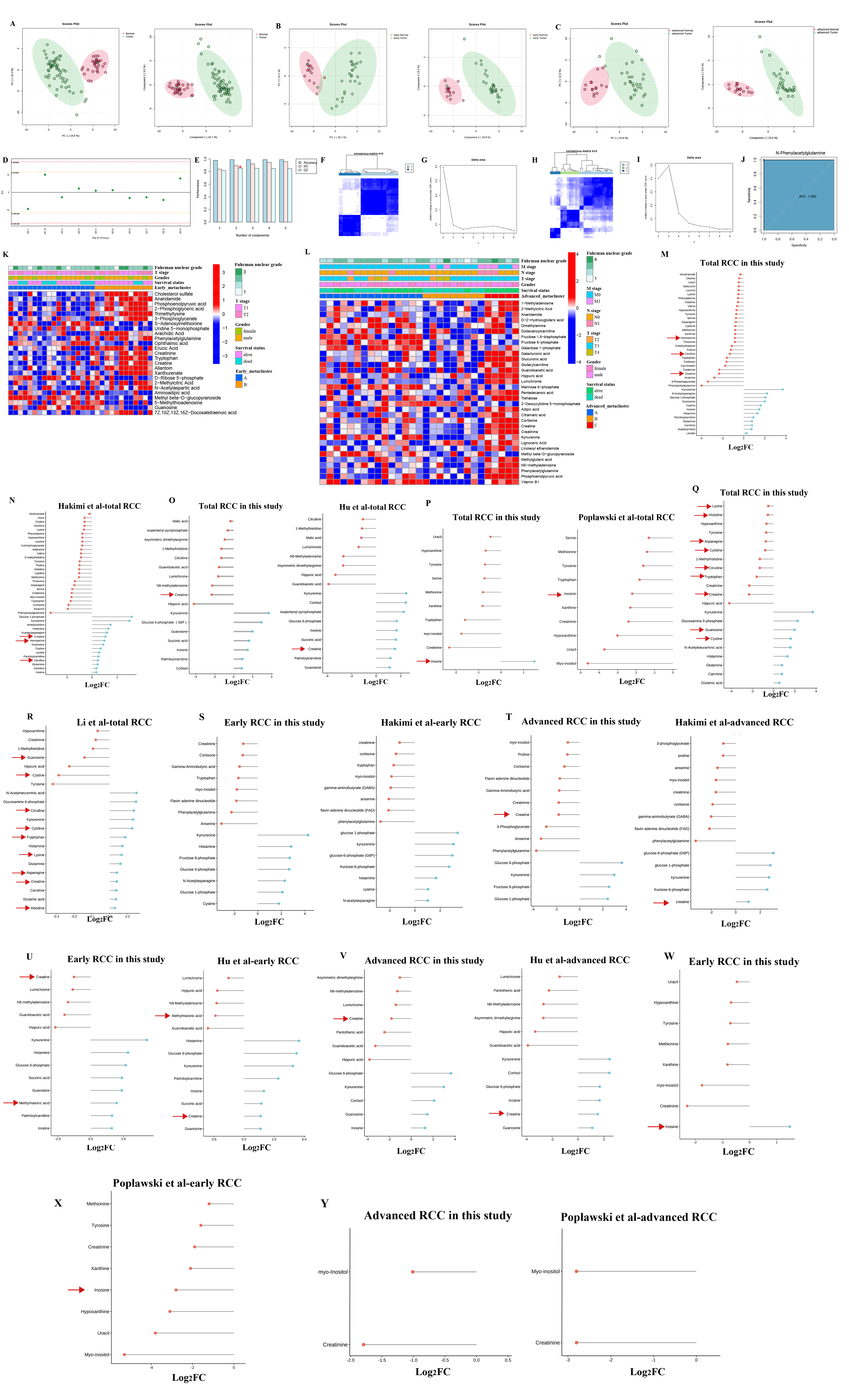

Supplement: Supplementary file 2 — FIGURE S1 (A) Score plot resulting from principal component analysis (PCA) (left) and PLS‐DA (right) analysis of metabolites in total patients. (B, C) Score plot resulting from PCA (left) and PLS‐DA (right) analysis of metabolites in patients with early‐stage (B) and advanced‐stage (C). (D) The PCA analysis of QC. (E) PLS‐DA cross‐validation plot based on all samples. (F–I) Unsupervised clustering analysis of differential metabolites identified in early‐stage samples (F, G) and advanced samples (H, I). (J) The ROC curve of N‐phenylacetylglutamine. (K, L) Unsupervised clustering of metabolites in early (K) and advanced (L) stage RCC cohort. The metabolic cluster, tumour T stage, survival status, gender, and Fuhrman nuclear grade were used as annotations. (M–R) The intersected differential metabolites in total RCC samples between this study and other independent studies. (S, U, W and X) The intersected differential metabolites in early‐stage RCC samples between this study and other independent studies. (T, V and Y) The intersected differential metabolites in advanced samples between this study and other independent studies. [file CTM2-14-e70142-s006.tif]
