# Supplementary material for: Identification of metabolic dysregulation and biomarkers for clear cell renal cell carcinoma
Source: Clin Transl Med. 2024 Dec 26;14(12):e70142. doi: 10.1002/ctm2.70142 (PMC11670740; doi:10.1002/ctm2.70142)
Supplement: Supplementary file 3 — TABLE S1 Characteristics of studies included in this study. [file CTM2-14-e70142-s001.docx]

**Supplementary Table 1.** Characteristics of studies included in this study.

| **First author** | **Publication year** | **Nation** | **Metabolome Analysis** | **Survival profile** | **Median follow-up** | **Numbers of samples** | **Treatments** | **Data source** |
| --- | --- | --- | --- | --- | --- | --- | --- | --- |
| Hakimi Ari A | 2016 | American | LC/MS and GC/MS | OS | 60 months | 138 | No | GEO: GSE74734 |
| Li Yize | 2023 | American | ESI-LC-MS/MS | OS | 689 days | 57 | No | Proteomic Data Commons ID: CPTAC3 |
| Hu junyi | 2024 | China | LC/MS and GC/MS | N/A | N/A | 100 | No | GSA-Human database PRJCA014547 |
| Piotr Popławski | 2016 | European | GC-MS | N/A | 43.22 months | 35 | No | Supplementary file |
| Li haoxin | 2019 | American | LC-MS | OS | 22.07 months | 91 | Yes (Nivolumab) | metabolomicsworkbench ID: PR000828. |
| Reigle james | 2021 | American | LC-MS | N/A | N/A | N/A | No | Supplementary file |

OS: overall survival.
