# Supplementary material for: Identification of metabolic dysregulation and biomarkers for clear cell renal cell carcinoma
Source: Clin Transl Med. 2024 Dec 26;14(12):e70142. doi: 10.1002/ctm2.70142 (PMC11670740; doi:10.1002/ctm2.70142)
Supplement: Supplementary file 4 — TABLE S2 Descriptive baseline characteristics of 60 patients diagnosed with clear cell carcinoma between 2020 and 2021. [file CTM2-14-e70142-s007.docx]

**Supplementary Table 2.** Descriptive baseline characteristics of 60 patients diagnosed with clear cell carcinoma between 2020 and 2021.

| **characteristics** | **ccRCC patients N (%)** |
| --- | --- |
| ***Baseline Characteristics*** |  |
| N | 60(100%) |
| Age (mean, y) | 57.77±1.142 |
| Smoking status |  |
| Yes | 6(10.0%) |
| No | 54(90.0%) |
| Alcohol consumption |  |
| Yes | 12(20.0%) |
| No | 48(80.0%) |
| Gender |  |
| Female | 19(31.7%) |
| Male | 41(68.3%) |
| Diabetes |  |
| Yes | 13(21.7%) |
| No | 47(78.3%) |
| Heart disease |  |
| Yes | 13(21.7%) |
| No | 47(78.3%) |
| BMI | 23.82±0.501 |
| ***Diagnostic characteristics*** |  |
| Waist pain |  |
| Yes | 11(18.3%) |
| No | 49(81.7%) |
| Blood urine |  |
| Yes | 16(26.7%) |
| No | 44(73.3%) |
| Tumor location |  |
| upper pole | 24(40.0%) |
| lower pole | 36(60.0%) |
| ***RCC with inferior vena cava thrombus*** |  |
| Yes | 26 (43.3%) |
| No | 34(56.6%) |
| ***Clinical TNM stage*** |  |
| Clinical T stage |  |
| cT1 | 15(25.0%) |
| cT2 | 19(31.7%) |
| cT3 | 23(38.3%) |
| cT4 | 3(5.0%) |
| Clinical N stage |  |
| N0 | 55(91.6%) |
| N1 | 5(8.3%) |
| Clinical M stage |  |
| M0 | 54(90.0%) |
| M1 | 6(10.0%) |
| Pathology |  |
| Clear cell carcinoma | 60(100%) |
| Furhrman nuclear grade |  |
| 1 | 5(8.3%) |
| 2 | 32(53.3%) |
| 3 | 21(35.0%) |
| 4 | 1(1.7%) |
| Hospital stay (mean, day) | 12.58±0.668 |
| Follow-up (median, month) | 16.2 (14.8-18.3) |
| Surgical methods |  |
| Laparoscopic surgery | 43(71.7%) |
| Robot-assisted laparoscopic surgery | 17(28.3%) |

ccRCC, clear cell carcinoma.
